# Inherently Analog Quantity Representations in Olive Baboons (*Papio anubis*)

**DOI:** 10.3389/fpsyg.2013.00253

**Published:** 2013-05-02

**Authors:** Allison M. Barnard, Kelly D. Hughes, Regina R. Gerhardt, Louis DiVincenti, Jenna M. Bovee, Jessica F. Cantlon

**Affiliations:** ^1^Department of Brain and Cognitive Sciences, University of RochesterRochester, NY, USA; ^2^Department of Comparative Medicine, University of RochesterRochester, NY, USA; ^3^Seneca Zoological ParkRochester, NY, USA

**Keywords:** numerosity, primates, baboons, object-file, analog magnitude

## Abstract

Strong evidence indicates that non-human primates possess a numerical representation system, but the inherent nature of that system is still debated. Two cognitive mechanisms have been proposed to account for non-human primate numerical performance: (1) a discrete object-file system limited to quantities <4, and (2) an analog system which represents quantities comparatively but is limited by the ratio between two quantities. To test the underlying nature of non-human primate quantification, we asked eight experiment-naive olive baboons (*Papio anubis*) to discriminate between number pairs containing small (<4), large (>4), or span (small vs. large) numbers of food items presented simultaneously or sequentially. The prediction from the object-file hypothesis is that baboons will only accurately choose the larger quantity in small pairs, but not large or span pairs. Conversely, the analog system predicts that baboons will be successful with all numbers, and that success will be dependent on numerical ratio. We found that baboons successfully discriminated all pair types at above chance levels. In addition, performance significantly correlated with the ratio between the numerical values. Although performance was better for simultaneous trials than sequential trials, evidence favoring analog numerical representation emerged from both conditions, and was present even in the first exposure to number pairs. Together, these data favor the interpretation that a single, coherent analog representation system underlies spontaneous quantitative abilities in primates.

## Introduction

From Euclid, who said, “The laws of nature are but the mathematical thoughts of God,” to the modern mathematical scholar Paul Dirac who stated, “If there is a God, he’s a great mathematician,” great thinkers have often associated abstract numerical thought with the divine. However, in contrast to human intuitions, cognitive science has demonstrated that the seemingly supernatural human capacity for symbolic mathematical thought – responsible for scientific measurement, architectural design, and economic exchange – likely arises from a primitive number representation system (or perhaps systems) that appear in creatures like beasts and babies. Evolutionarily and developmentally primitive numerical systems are well-documented. Non-linguistic infants can reason about small and large numerosities (for reviews on the extensive literature, see Feigenson et al., 2004; Cordes and Brannon, 2008), as can many non-human animals (Primates: e.g., Brannon and Terrace, 1998, 2000; Hauser et al., 2000; Beran and Rumbaugh, 2001; Cantlon and Brannon, 2006, 2007; Other mammals: e.g., Jaakkola et al., 2005; Ward and Smuts, 2007; Uller and Lewis, 2009; Birds: e.g., Pepperberg, 2006; Rugani et al., 2007, 2008, 2010; Amphibians: e.g., Uller et al., 2003; Fish: e.g., Agrillo et al., 2007). However, the precise nature of the representations underlying infant vs. animal quantity judgments has been a subject of discussion in the numerical cognition literature.

Non-linguistic numerical cognition in human infants is hypothesized to involve two different mechanisms: a precise object-file system and an analog magnitude system. The object-file system is thought to be an aspect of working memory, which individuates, enumerates, and tracks objects, and so, is inherently capable of tracking the number of objects (Trick and Pylyshyn, 1993, 1994). As working memory is limited to tracking three or four objects, the signature of the object-file system as a number representation system is the failure of an individual to discriminate between two quantities if at least one of those quantities is larger than three (or four). The analog system also has a set of signatures that can be used to detect its functioning (Dehaene, 1997). Unlike the object-file system, the analog system is (in principle) capable of representing any number. Instead of bearing a capacity limit, the analog system is limited by the ratio of two compared quantities, with crude ratios being more distinguishable than fine ratios. For example, two numbers that have a crude ratio, such as 1 and 4 (0.25 ratio) will be easily discriminated, while two numbers with a fine ratio, such as 3 and 4 (0.75 ratio), may not be discriminated. The magnitudes are psychologically spaced either logarithmically or linearly with scalar variability, and because of this, the numerical ratio (and not the absolute numerical value) is the critical variable that determines whether two quantities can be discriminated in the analog system (e.g., Gallistel and Gelman, 1992; Cantlon et al., 2009). The proportion difference in quantity needed to successfully discriminate between two quantities is called the Weber fraction. In summary, the analog system is limited by ratio but provides a larger range of numerical values that can be represented whereas the object-file system is precise, but limited to representing only very small quantities.

The existences of the object-file system and the analog system are not controversial. The role of the object-file system in tracking objects is well established. The relevant research questions are if, when, and how the object-file system is spontaneously recruited to represent quantity. Infants appear to use both an analog and an object-file system to compare quantities spontaneously. With small numbers of objects (<4), infants are capable of making correct numerical judgments no matter the ratio between sets (Feigenson et al., 2002; Feigenson and Carey, 2003). In contrast, when infants judge larger sets, accurate discrimination is a function of ratio (Xu and Spelke, 2000; Xu, 2003; Lipton and Spelke, 2004; Xu and Arriaga, 2007). Most importantly, studies have found that when infants and toddlers are required to compare a small vs. large set on a given trial, their performance is random (Feigenson and Carey, 2005; Le Corre and Carey, 2007; but see Cordes and Brannon, 2009; Cantlon et al., 2010). Researchers argue that the comparison of a small and large value cannot be completed because each numerical system handles just one type of number (small or large) and the two systems do not communicate (Feigenson et al., 2002; Feigenson and Carey, 2003). The finding of failures of infants to compare small and large numbers is taken as further evidence of the presence of two distinct numerical systems.

The pattern of success and failure observed in infants is not observed in adults. When adults are asked to judge quantities while their verbal abilities are occupied by an articulatory suppression task, their number discrimination behavior exhibits the ratio signature of the analog system for small and large numbers alike (Whalen et al., 1999; Cordes et al., 2001; Barth et al., 2003; Beran et al., 2006). Adults can successfully discriminate small (<4) from large (>3) sets in the same comparison, unlike infants. Likely, the analog system is the primary non-linguistic number representation system in adults, although the object-file mechanism might be recruited for quantitative judgments under limited conditions (Trick and Pylyshyn, 1993, 1994). Current evidence suggests that the adult pattern of predominantly analog numerical representation emerges by at least 3 or 4 years of age (Halberda and Feigenson, 2008; Cantlon et al., 2010).

The data from human infants and adults raise the question of whether the “two systems” view of quantity development also characterized the evolution of numerical cognition. Comparative studies of numerical cognition with non-human animals have yielded mixed results on the fundamental nature of number cognition in non-humans. In one study, Hauser et al. (2000) presented experiment-naive rhesus macaques with differing quantities of apple slices dropped sequentially into two boxes. The macaques chose the box with the larger amount in 1 vs. 2, 2 vs. 3, 3 vs. 4, and 3 vs. 5 contrasts, but failed with larger numbers such as 4 vs. 5 and span contrasts such as 3 vs. 8. Given these data, these researchers concluded that only the object-file system is spontaneously available to rhesus macaques, a position that was supported by subsequent testing with the same population (Hauser and Carey, 2003; Barner et al., 2008; Wood et al., 2008). However, the majority of other non-human number studies have not upheld these results. For instance, Beran (2007) found evidence of the analog system in rhesus macaques with a joystick task that was highly similar to Hauser’s design. Subjects watched as an image of a hand appeared to drop between 1 and 10 blocks into two boxes onscreen. Macaques succeeded at choosing the box with the larger quantity of blocks at above chance levels with both small and large set sizes, with success on each contrast being a function of numerical ratio. In critical test trials, Beran’s macaques also succeeded when contrasts involved one small set (<4) and one large set (>4), suggesting that a single-system, the analog system, was responsible for all number representation in those macaques. Other numerical studies have also supported the analog magnitude hypothesis in multiple primate species (Brannon and Terrace, 1998, 2000; Beran and Rumbaugh, 2001; Smith et al., 2003; Beran and Beran, 2004; Judge et al., 2005; Brannon et al., 2006; Cantlon and Brannon, 2006, 2007) using a variety of testing paradigms. Those data also implicate the analog magnitude system as the core mechanism of number representation in primates.

Growing evidence suggests that the analog magnitude system is the evolutionarily primitive number system shared across animal lineages. Less clear is whether the analog magnitude system is the primary mechanism that non-human animals recruit *spontaneously* to solve quantity problems. There is already some evidence that apes spontaneously show ratio effects in their numerical performance (Beran, 2001, 2004; Beran and Beran, 2004; Hanus and Call, 2007). Although those studies did not compare the animals’ performance on small vs. large values during first exposures, the rapid emergence of ratio effects in performance supports the analog magnitude hypothesis. Currently, most studies concluding that primates primarily rely on the analog magnitude system have been conducted with subjects that have long histories of exposure to experimental methods (e.g., Brannon and Terrace, 1998; Smith et al., 2003; Cantlon and Brannon, 2006, 2007; Beran, 2007; Beran et al., 2008). It is sometimes argued that such exposure could influence the strategies that subjects recruit to solve problems. For example, Hauser et al. (2000) argued that experiments that involve extensive training could allow animals to acquire the cognitive abilities necessary for solving numerical tasks. In the Beran (2007) study described above, the subjects had participated in several previous numerical cognition studies where they received thousands of trials of experience with numerical discriminations. Thus, there remains the possibility that the training period associated with the laboratory experiments caused the discrepancy in results between the laboratory experiments (e.g., Beran, 2007) and the naturalistic experiments (Hauser et al., 2000).

In the current study, we tested quantity discrimination in experiment-naive olive baboons using a naturalistic food choice task that is similar to the Hauser et al. (2000) and Beran (2007) designs but without the extensive training regimen of Beran (2007). In the current experiment, baboons were presented with two sets of one to eight peanuts placed simultaneously or sequentially into two of three cups, and were rewarded with the contents of the cup they chose. The baboon subjects were experiment-naive, having never participated in psychological experiments. Together, these design elements allowed us to test for the spontaneous quantitative representations that baboons use naturally, during food choices.

In Experiment 1, we examined the spontaneous quantity representations of eight olive baboons by analyzing their quantity choices on first exposure to each number pair. Subjects compared numerical values in pairs of both small (<5), both large (>4), and span sets (one small and one large value). This range of sets allowed us to test the object-file and analog magnitude hypotheses. If the baboons are successful only with contrasts of small sets, then the object-file hypothesis will be supported. If the baboons are successful with small and large, but not span sets, we can conclude that both the object-file and the analog magnitude systems are engaged by baboons, as in human infants, but that the object-file and analog magnitude representations are incompatible resulting in failure on span sets. Finally, prior research with infants found that infants succeeded at discriminating large and span pairs in simultaneous but not sequential presentations (Feigenson et al., 2002). The argument is that the object-file mechanism is selectively recruited during sequential presentations due to its primary function as an object tracking system. Evidence for object-file-based quantity judgments in monkeys would thus include failures on both large and span pairs only for sequential presentations. However, if we find that our subjects can accurately make choices no matter the set size, and that accuracy on a particular numerical pairing is explained by numerical ratio, then we will have evidence that the analog magnitude system is the dominant mechanism for number representation in baboons, similar to human adults. In addition to testing these hypotheses, we were also interested in the effects of experience on primate number representation. In Experiment 2, we extended the testing of two baboons that participated in Experiment 1 for additional sessions, and analyzed changes in their sensitivity to numerical discriminations over time. The results we report aim to further our understanding of the fundamental nature of numerical representation in non-human animals.

## Experiment 1

### Materials and methods

#### Subjects

Eight adult olive baboons (4–14 years old, three male) at the Seneca Park Zoo in Rochester, NY, USA served as subjects in this experiment. These baboons are housed as a social group and have access to large indoor and outdoor enclosures. In addition, these enclosures have multiple compartments that allow us to temporarily segregate one baboon from the rest of the troop for testing purposes. Primate chow and fresh fruits and vegetables are provided every morning, and water is available *ad libitum*. Prior to these experiments, subjects had no experience with cognitive testing. The first experimental experience of these baboons was during the preparation phase of this experiment which trained subjects to choose the cup containing occluded food vs. two empty cups. In the preparation phase task, baboons were trained to use the apparatus by tracking a 1/2-grape hidden below one of three metal compote cups, and were rewarded for touching the port corresponding to the hidden food. Subjects required less than one session to choose the baited cup. Immediately following the preparation phase, the baboons were tested with the numerical food choice discriminations described below. Work with these subjects was approved by the Institutional Animal Care and Use Committee of the University of Rochester.

#### Apparatus

**Figure 1:**
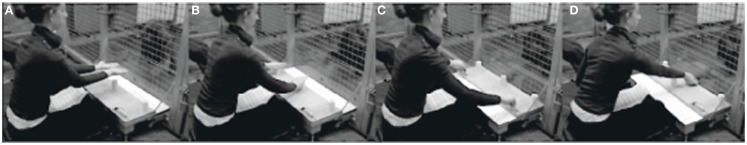
**The apparatus for Experiments 1 and 2**. The apparatus consisted of a short and small table, open on one side to the experimenter, and shielded by plexiglass on the subject’s side. The table was pushed flush with the subject’s enclosure for use. **(A)** To initiate a trial, the experimenter showed a subject a quantity of reward in her hand(s) (simultaneous condition shown). **(B)** The reward(s) were placed into opaque cylinders. **(C)** The panel was pushed forward and the subject indicated his choice by pointing to a cylinder. **(D)** The subject received the reward he selected.

The apparatus consisted of a small and short rectangular table (75 cm long × 35 cm deep × 17 cm high) that was a comfortable height for a seated baboon (Figure 1). One long side of the table top was open so that an experimenter could work the apparatus, but the other three sides were shielded by plexiglass (30 cm high) to prevent baboons from interacting with the apparatus until the appropriate time. When in use, the long side of plexiglass was pushed flush with the mesh of an enclosure, a subject sat behind the plexiglass (and the mesh of the enclosure), and an experimenter sat opposite the subject. There were three equally spaced ports (2.5 cm diameter) in the plexiglass that subjects could use to indicate their choices.

All experimental manipulations were conducted on a sliding panel (75 cm long × 17 cm deep) that sat atop the table. The purpose of this panel, which was the same length as the table, but only half as deep, was to control a subject’s access to the experiment until the appropriate time. When the panel was close to the experimenter, the subject did not have access to the experimental items, however, when the panel was pushed forward, toward the subject, the subject could reach through a port in the plexiglass and indicate her choice. The contents of the panel were three identical, opaque, cardstock cylinders, placed upright on a circular end, each in front of one of the ports in the plexiglass shield. The cylinders were open on both circular ends so that the experimenter could drop items into a cylinder and also lift a cylinder up, leaving the contents of a cylinder on the panel. Once items were dropped into the cylinders the items were hidden from a subject. The items to be enumerated were unshelled half peanuts.

### Procedure and design

#### General procedure

Each session was conducted by two experimenters. One experimenter worked the apparatus, while a second experimenter recorded the choices made by the subject, monitored the first experimenter for trial accuracy, and also operated a video camera which was used to record each session. Sessions were conducted when a subject could be temporarily isolated from the troop in an enclosure. Individuals were tested between one and three times a week.

Before testing began, the experimenters set up the apparatus: the plexiglass side of the table was placed flush with the subject’s enclosure, the sliding panel was placed on the experimenter’s side of the table, the three cylinders were set in place on the panel, and one experimenter sat opposite the subject. A trial could only be initiated if the subject was seated at and attentive to the apparatus. To initiate a trial, an experimenter showed the subject one or more peanuts; this was done by displaying peanuts in the palms of one or both hands, about 30 cm from the subject and above the experimental panel (Figure 1A). Half of all trials were *simultaneous condition* trials, in which the two number sets to be compared were presented simultaneously, one in each hand. For example, if the subject was to be tested on the contrast 3 vs. 6 items, the experimenter would have three peanuts in the palm of one hand and six peanuts in the palm of the other hand. The experimenter showed these options to the subject for approximately 2 s, after which the experimenter simultaneously placed the contents of each hand into its own cylinder (Figure 1B). In doing so, the experimenter touched both cylinders in the same way and for an equal amount of time. The entire process of peanut selection, presentation, and placing it in a cylinder took approximately 4 s. The remaining trials were the *sequential condition* trials, in which only one peanut was presented at a time. Each peanut was presented to the subject for approximately 2 s and then placed into a cylinder. For example, if the experimenter was testing the numerical pair 3 vs. 6, the first three peanuts presented were baited into one cylinder one-at-a-time, and the following six peanuts were then baited into a second cylinder one-at-a-time. Again, the experimenter was careful to touch the cylinders in the same way and for the same amount of time. To ensure that subjects were not basing their choices on the spatial location of the sets, the larger and smaller numerical values were equally likely to appear in any one of the three cylinders across the session. Note, that although there were always three cylinders on the board, only two of these cylinders were baited with food on each trial. The presence of the third cylinder allowed us to monitor subjects’ understanding of the general task requirement that only baited cups should be chosen. Subjects almost never selected the empty cylinder (4% of sequential and 3% of simultaneous trials) indicating that they understood the task.

After the cylinders had been baited with peanuts, the panel was pushed forward and the subject was allowed to make a choice from among the three cylinders (Figure 1C). Experimenters did not look at the cups after baiting until after the subject indicated their choice. The subject indicated its choice by poking its finger through the port in front of the desired cylinder. Then, the experimenter removed the cylinder from over the desired food, and the food reward was fed, one peanut at a time, to the subject through the same port (Figure 1D). In the case that there was no food reward under the chosen cylinder, the subject received no reward. When the subject had received the entirety of its reward, the experimenter removed the other two cylinders from the panel, revealing their contents. The experimenter removed the reward not chosen. Once all peanuts were removed from the board, the experimenter pulled the panel back to her side of the apparatus, and reset the board. The next trial was initiated. This procedure was used throughout the training and testing phases.

#### Training

In the training phase, we exposed the subjects only to the numerical comparison 1 vs. 2, presented both simultaneously and sequentially. Subjects were given multiple sessions until they chose the larger reward set at above chance levels within a single session as determined by a cumulative binomial analysis (threshold = 24/36 correct). Each session consisted of 36 trials; these trials were counterbalanced for baiting locations, simultaneous vs. sequential conditions, and in the case of sequential trials, for which number set was baited first. Progress through the session was closely monitored. If a gap of 5 min occurred between two trials due to subject inattention, the session was terminated, and training resumed the next time the subject was available. Terminated sessions were rare and excluded from analyses. Once the subject passed the training criterion they immediately began the testing phase of the experiment. Subjects needed 1.5 sessions on average (54 trials) to reach our criterion.

#### Testing

Testing was conducted over 54 total test trials, across two 30-min sessions. The 27 different number pairs ranging from 1 to 8 items were tested (all possible pairs excluding 1 vs. 2 which was the training pair), with each number pair tested once in sequential and once in simultaneous presentation. The beginning of each testing session consisted of a warm-up of four 1 vs. 2 trials (two simultaneous, two sequential) to ensure the subject was oriented to the task. Two additional trials of 1 vs. 2 (one sequential, one simultaneous) were tested in each session but those trials were not analyzed as 1 vs. 2 was the training and warm-up pair. If the subject failed more than half of these first trials, testing with that subject was terminated for the day. The order of the test trials was randomized within and between subjects. Also, as in training, baiting locations, simultaneous vs. sequential conditions, and in the case of sequential trials, which baited first, were randomized. In addition, pair size, presentation type, and location of the larger quantity were never repeated on more than three consecutive trials. If a gap of 5 min occurred between two trials due to subject inattention, the session was stopped, and the remaining trials were resumed after a warm-up during the next testing day.

#### Data analysis

Data were coded and analyzed by an independent observer who coded responses from the recorded video files. Weber fractions were calculated using methods reported in Cantlon and Brannon (2006).

### Results

#### Training

Subjects took an average of 1.5 sessions to reach the training criterion of above-chance performance within a single session on 1 vs. 2 numerical comparisons according to a binomial test (range = 1–2 sessions).

#### Testing

**Figure 2:**
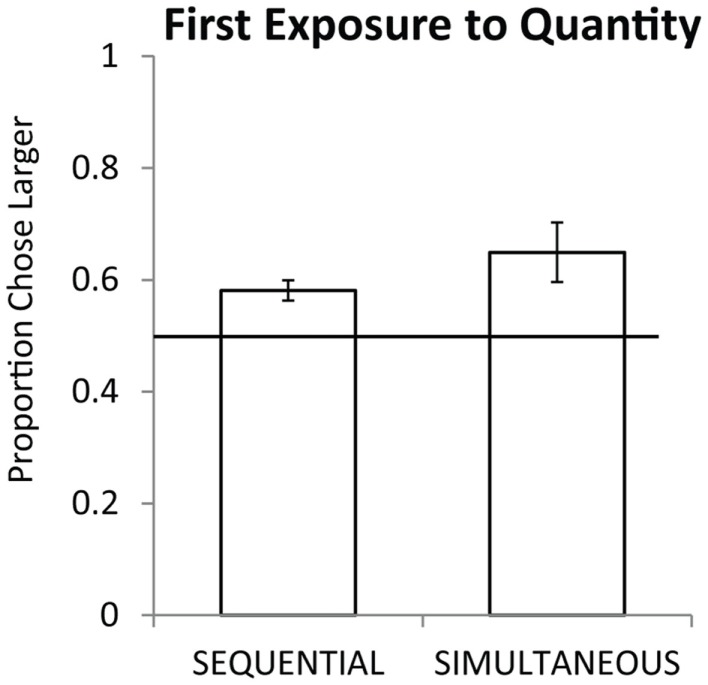
**Average accuracy for eight monkeys on sequential and simultaneous trials from their first exposure to numerical discriminations**. Chance is a 0.5 probability. Error bars represent the standard error of the mean across monkeys.

**Figure 3:**
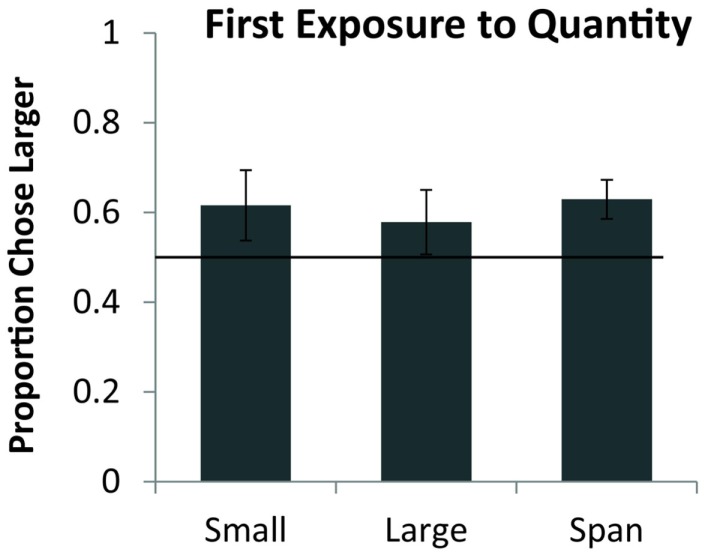
**Average accuracy for eight monkeys on the small (both numbers <5), large (both numbers >4), and span pairs (one number <5 and one number >4) on their first exposure to numerical discrimination**. Chance is a 0.5 probability. Error bars represent the standard error of the mean across monkeys.

Seven monkeys completed all 54 trials of testing, the eighth completed 21 of 27 sequential trials and 22 of 27 simultaneous trials. As a group, monkeys preferentially selected the larger quantity on the first exposure for simultaneous pairs [chance = 50%, one sample *t*(7) = 2.76, *p* < 0.05] and for sequential pairs [one sample *t*(7) = 4.38, *p* < 0.01]. Figure 2 shows the overall performance on simultaneous and sequential trials. Simultaneous performance was marginally higher than sequential performance across the group but the difference was not significant [*t*(7) = 1.74, *p* = 0.12]. Figure 3 shows performance on the three pair types tested: small, large, and span pairs. Monkeys performed significantly above chance on small number pairs [*t*(7) = 3.21, *p* < 0.05] and span pairs [*t*(7) = 2.99, *p* < 0.05] but large pair performance was non-significant [*t*(7) = 1.44, *p* = 0.19]. Poor performance on large numbers could be explained by the fact that large number pairs have inherently more difficult discrimination ratios. Further analyses revealed that on the sequential trials, particularly for large numbers, monkeys had a bias to select the more recently presented food set [*t*(7) = 5.17, *p* < 0.01]. This bias on large sequential trials cannot explain successful performance on the other pair types because the larger number was equally likely to be presented first or second on sequential trials and on simultaneous trials both sets were presented at the same time. Instead, performance across all number pairs was predicted by numerical ratio.

**Figure 4:**
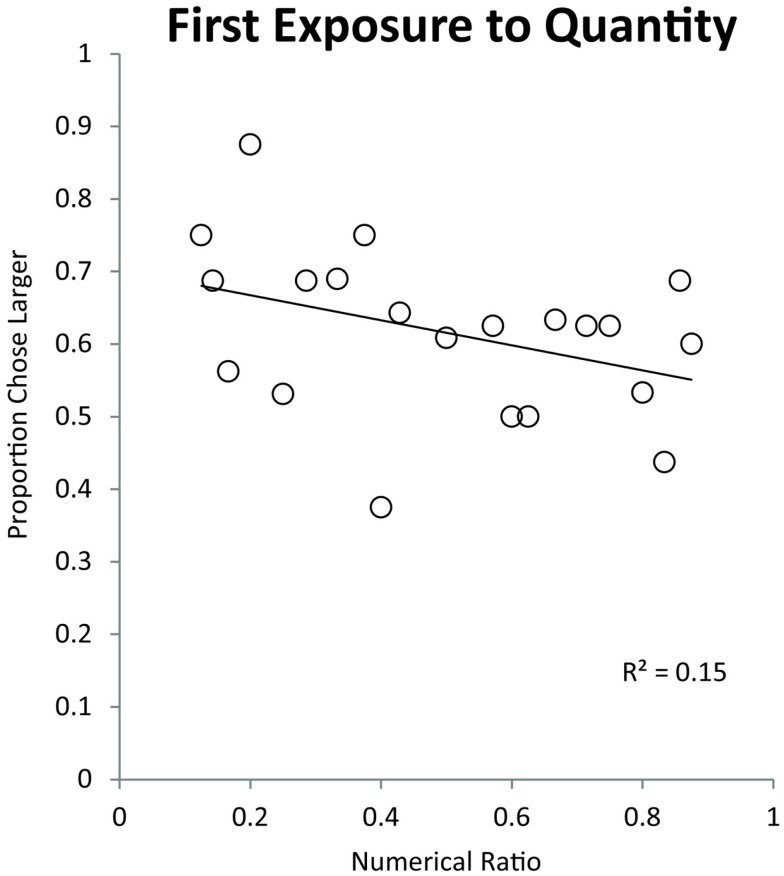
**Effect of numerical ratio on monkeys’ accuracy during their first exposure to numerical discrimination**. The numerical ratio is the smaller number in a given pair divided by the larger number. Each data point represents a different number pair. The trend line represents a linear trend of decreasing accuracy with finer numerical ratios.

We binned the numerical pairs by their numerical ratio and tested for a linear trend of numerical ratio. Monkeys showed a significant effect of ratio on their simultaneous trial performance indicating that they made choices on the basis of an analog quantity representation [Pearson’s *r*(19) = −0.38, *p* < 0.05]. This finding suggests that monkeys struggled to discriminate large values in part due to their more difficult discrimination ratios. Figure 4 shows the effect of ratio on monkeys’ quantity discrimination in this first exposure task. Performance on numerical pairs ranges from approximately 75% on easy ratios to 55% on difficult ratios.

We calculated individual Weber fractions for the five animals that performed above chance overall on the first exposure task. Weber fractions on first exposure ranged from 0.51 to 0.91, which is comparable to the range of Weber fractions exhibited by young children on similar tasks (Halberda and Feigenson, 2008).

## Experiment 2

### Methods and materials

#### Subjects

Subjects for Experiment 2 were two female baboons who participated in Experiment 1 (Pearl, Ursala).

#### Apparatus, procedure, design, and data analysis

In Experiment 2 we collected more data on numerical comparisons from two subjects in order to detect subtle performance signatures among pair types. We used the same apparatus and procedure as in Experiment 1. Three cups were presented on each trial as choices, two of the cups were baited. In this experiment, monkeys never chose the empty cup. The numerical values presented ranged from 1 to 8. Each session began with a five-trial 1 vs. 2 “warm-up.” The test immediately followed the warm-up and contained approximately eight of each small, large, and span test pairs (four each of simultaneous and sequential) and four 1 vs. 2 trials randomly interspersed throughout the test trials. Sessions were equated for mean numerical ratio across conditions. The numerical ratio was an average of approximately 0.5 for each pair type in each session. This meant that some pair types were tested more frequently than others. We tested the Experiment 2 subjects until they had multiple exposures to all contrasts in both presentation types. We completed 11 sessions with Pearl and 13 sessions with Ursala, as described in the procedure for Experiment 1. Extra sessions were required due to incomplete trials during some sessions. Ultimately, each animal completed approximately 130 trials per presentation format. There was a total average of approximately 4.5 trials per number pair, per presentation format.

It is important to note that the animals were not trained to select the larger quantity over the course of time. Instead, animals were always rewarded with the cache that they chose. The only way that a longer exposure period could possibly result in learning over time is if the animals are able to discriminate the quantities of the choices they were given. That is, because the animal was rewarded with their chosen food quantity on each trial, in order to learn that the quantity they chose was either greater or lesser than the alternative quantity, they would have to be able to discriminate and compare the quantities of the chosen vs. unchosen rewards.

### Results

**Figure 5:**
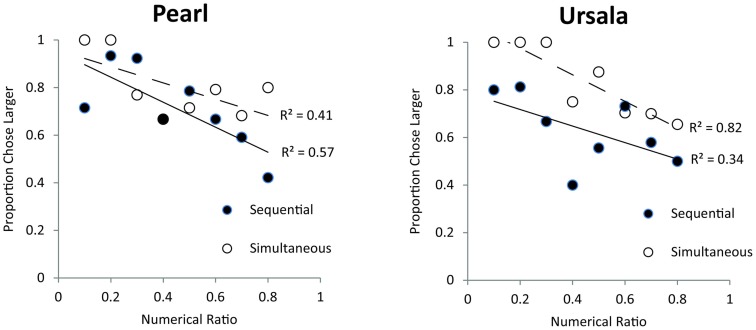
**Effect of numerical ratio on accuracy from the two monkeys (Pearl and Ursala) tested in the longer exposure (Experiment 2) for sequential (filled circles) and simultaneous trials (open circles)**. Trend lines represent linear trends of decreasing accuracy with finer numerical ratios.

In this longer exposure task, both monkeys significantly chose the larger food set for both sequential trials [binomial tests, Pearl (83/120 trials), *p* < 0.001, Ursala (91/141 trials), *p* < 0.001] and simultaneous trials [Pearl (96/121 trials), *p* < 0.001, Ursala (114/141 trials), *p* < 0.001]. These data indicate that animals can spontaneously discriminate quantities presented simultaneously and sequentially. Additionally, these monkeys were successful at choosing the larger quantity for small pairs [binomial test, Pearl (62/80 trials), *p* < 0.001, Ursala (63/85 trials), *p* < 0.001], span pairs [Pearl (64/80 trials), *p* < 0.001, Ursala (85/102 trials), *p* < 0.001], and large pairs [Pearl (53/81 trials), *p* < 0.01, Ursala (57/95 trials), *p* < 0.05]. This above-chance performance for small, span, and large quantity pairs supports the conclusion that olive baboons spontaneously use the analog numerical system to solve this task. The only exception is that for large sequentially presented pairs monkeys displayed a bias in selecting the most recently presented set [binomial test on choosing the second cache, Pearl (27/40 trials), *p* < 0.05, Ursala (43/45 trials), *p* < 0.001; all *p*’s for other five pair types >0.05]. However, since monkeys’ performance was significantly above chance on sequentially presented span pairs, there is still evidence that spontaneous analog quantity representations were used to solve the sequential task [Sequential span pairs: Pearl (29/40 trials), *p* < 0.01, Ursala (39/52 trials), *p* < 0.001]. In addition, both monkeys showed a ratio effect across both the simultaneous and sequential trials, implicating the analog numerical system [Simultaneous pairs: Pearl: *r*(19) = −0.43, *p* < 0.05; Ursala: *r*(19) = −0.59, *p* < 0.01; Sequential pairs: Pearl: *r*(19) = −0.66, *p* < 0.001; Ursala: *r*(19) = −0.36, *p* = 0.05]. Figure 5 shows performance for each monkey, for each pair type as a function of numerical ratio. Each data point on Figure 5 represents a different numerical pair. The individual Weber fractions for both monkeys over longer exposure were 0.44. This fraction is slightly better than Pearl’s Weber fraction on the first exposure task (0.57) and comparable to Ursala’s (0.39).

#### Comparison of monkey performance to predictions from the object-file hypothesis

**Figure 6:**
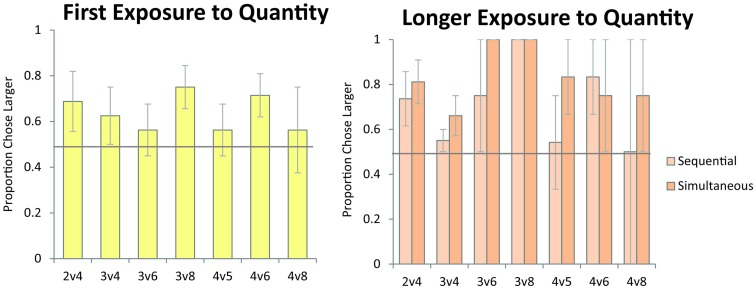
**Monkeys’ accuracy on numerical pairs which have been previously reported to elicit failures under the object-file system**. In contrast to the predictions of the object-file model, monkeys performed significantly above chance across these pairs on their first exposure to numerical discrimination (left panel, data from Experiment 1) and on longer exposure to the numerical task (right panel, data from Experiment 2) for both the sequential and simultaneous conditions. Error bars represent standard error of the mean across monkeys. Chance is a 0.5 probability.

**Figure 7:**
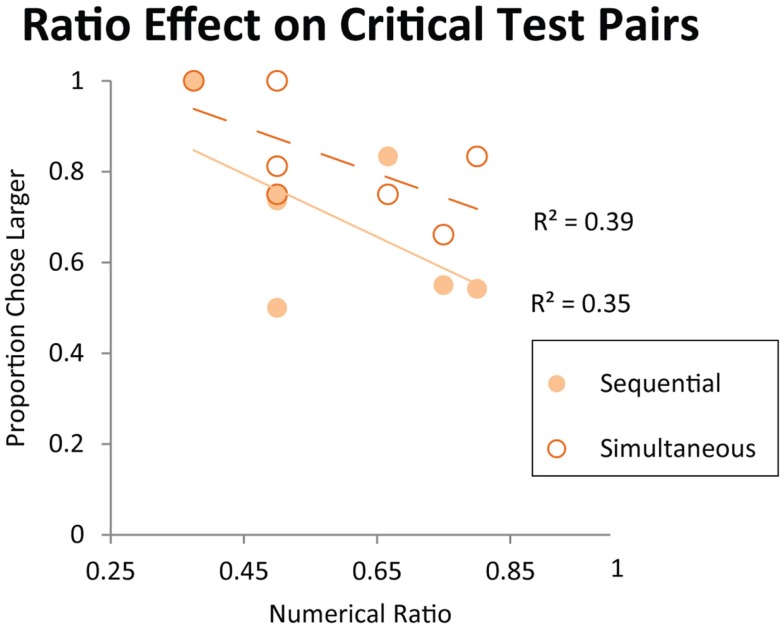
**Performance on the critical test pairs for object-file representation: 2 vs. 4, 3 vs.4, 3 vs. 6, 3 vs. 8, 4 vs. 5, 4 vs. 6, 4 vs. 8**. All of these pairs have been reported as failures in monkeys, infants, or both and those failures have been cited in support of the object-file hypothesis (Hauser et al., 2000; Feigenson et al., 2002). Each data point on the graph represents a different numerical pair from this set. Trend lines represent linearly decreasing accuracy as numerical ratio gets finer. Contrary to the predictions of the object-file hypothesis, accuracy on these numerical pairs is modulated by numerical ratio in monkeys.

As discussed in the Introduction, failure on numerical pairs with one value greater than three has been taken as evidence for object-file representation in the literature on infant quantity development (Feigenson et al., 2002). In order to test whether monkeys exhibit the same patterns of performance as human infants, we tested monkeys’ performance on specific pairs that human infants have been shown to fail at in previous studies. Here, we examined the data from Experiment 1 (first exposure) and Experiment 2 (longer exposure). We tested the numerical pairs 2 vs. 4, 3 vs. 4, and 3 vs. 6, which human infants fail to discriminate. We also tested monkeys’ performance on numerical pairs that were previously argued to elicit discrimination failures in monkeys, and were argued to provide evidence of object-file quantity representation in non-human primates (Hauser et al., 2000). Figure 6 shows monkeys’ performance on all numerical pairs which monkeys would be predicted to fail to discriminate under an object-file hypothesis: 2 vs. 4, 3 vs. 4, 3 vs. 6, 3 vs. 8, 4 vs. 5, 4 vs. 6, 4 vs. 8. Again, all of these pairs have been reported as failures in monkeys, infants, or both and those failures have been cited in support of the object-file hypothesis (Hauser et al., 2000; Feigenson et al., 2002). In contrast to prior infant and some non-human primate findings, monkeys performed significantly above chance on these pairs, even on their first exposure in Experiment 1 [First Exposure Group: one sample *t*-test vs. chance, *t*(7) = 2.99, *p* < 0.05; Longer Exposure Group: Binomial Tests vs. chance, Sequential: Pearl (18/27, *p* = 0.06), Ursala (23/32, *p* < 0.01], Simultaneous: Pearl (22/27, *p* < 0.001), Ursala (25/32, *p* < 0.01). Moreover, seven out of eight monkeys in Experiment 1 performed above 50% on these critical test pairs on the first trial (Binomial test 7/8, *p* < 0.05). Instead of failing to discriminate pairs in which one quantity exceeded the object-file capacity limit of three or four, performance on these pairs was modulated by ratio for both the simultaneous and sequential presentations implicating analog magnitude representations of number (Figure 7). The presence of a ratio effect on these critical test pairs indicates that analog magnitude representations were used to compare these quantities.

Finally, in order to confirm that the animals were not learning these “predicted fail pairs” over time, we tested for trends of improving accuracy across the longer exposure period for the predicted fail pairs. Neither monkey showed a significant improvement in accuracy as a function of time for these pairs [Pearl *r*(11) = 0.31, *p* = 0.35; Ursala *r*(13) = −0.04, *p* = 0.90].

#### Control condition

In Experiments 1 and 2 there is a possibility that animals used subconscious cues from the human experimenters to solve the task. This possibility seems unlikely for several reasons. First, we found evidence that the animals selected the larger quantity on their first exposure to the task and did not learn the task by trial-and-error, contrary to accounts of human cueing which are hypothesized to require associative learning over the course of training (i.e., Clever Hans; see Beran, 2012). Secondly, as mentioned earlier, in order for these animals to engage in associative learning during our food choice task they would have to be able to discriminate the quantity of the reward they received from the quantity that they did not receive. This is because the animals are rewarded on every trial with some quantity of food: either the larger or the smaller amount. If the animals have the spontaneous quantitative abilities to discriminate the reward they received from the one they didn’t, then they would likely use those spontaneous quantitative abilities to choose the larger number of food items rather than using their quantitative abilities to arduously form associations between the relatively larger quantity and human cues. Third, the monkeys’ performance was modulated by ratio whereas the human experimenters knew precisely the quantity of food items in each cup and thus would have subconsciously cued precise performance by the baboons. In short, it seems unlikely that human cueing played a role in the animals’ quantity decisions. Unfortunately the literature on primates’ abilities to use subtle human body language cues is sparse and so, it is unclear what behavioral patterns we should expect to see if the animals in our experiments used subconscious human cues to guide their choices. The main source of data on this issue is the Clever Hans phenomenon, a horse who (it is claimed) used the tension in the body language of his trainer to accurately respond to mathematical tasks. Currently, there are no rigorous experimental data that show whether, when, or how non-human primates are able to use such cues spontaneously or otherwise to guide decisions. And so, in order to rule out the possibility that our animals used subconscious cues from human experimenters to guide their food choices, we conducted a controlled test.

## Methods and Materials

### Subjects

Subjects were the two female baboons who participated in Experiment 2 (Pearl, Ursala).

### Apparatus, procedure, design, and data analysis

In the control condition, the two animals from Experiment 2 were tested by two experimenters, each of whom baited one of the two cups. Each experimenter was blind to the quantity of food items baited into the other’s cup and so was unaware of which cup contained the larger quantity. This ensured that the human experimenters could not give subconscious cues to the correct cup because they did not know which cup was correct. Monkeys were tested with approximately 55 trials of the number pairs 1 vs. 2 and 2 vs. 9 in the sequential presentation format. Each session was 24 trials. The procedure was otherwise identical to Experiments 1 and 2.

## Results

**Figure 8:**
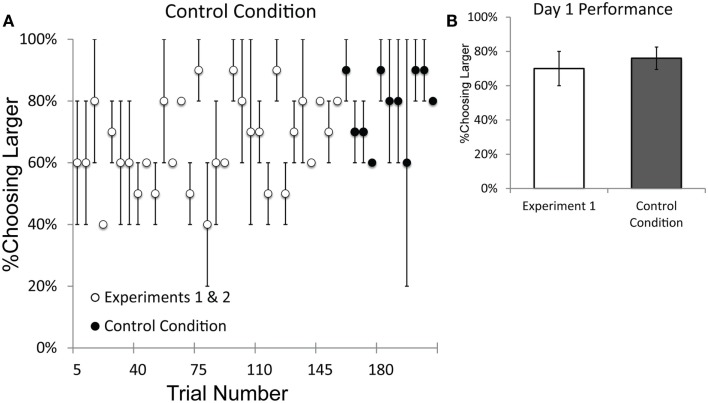
**Performance on the Control Condition, which controlled for the possibility of subconscious cueing by experimenters**. **(A)** Performance on the sequential trials of Experiments 1 and 2 and the Control Condition, plotted by trial number. Performance on the Control Condition was not impaired relative to performance on Experiments 1 and 2. **(B)** A comparison of performance on the first day of testing for Experiment 1 vs. the first day of testing on the Control Condition. The data presented from Experiment 1 only include numerical pairs that are as easy as those from the Control Condition (numerical ratios ≤0.5). Error bars represent standard error of the mean difference between monkeys.

Both monkeys performed significantly above chance from the first session of testing with the control condition (Binomial tests; Pearl: 19/24, *p* < 0.01; Ursala: 17/24, *p* < 0.05). Figure 8A plots the data in five-trial increments from the beginning of testing and illustrates that performance on the control condition was comparable to performance from Experiments 1 and 2. A *t*-test comparing the first 11 blocks of Experiments 1 and 2 with the 11 blocks of the control condition showed no decrement in performance on the control condition [Experiments 1 and 2: 61%; Control: 78%; *t*(10) = 4.03, *p* = 0.002]. In fact, performance on the control condition was slightly better overall than performance in Experiments 1 and 2. Figure 8B illustrates that there was no difference in performance between the first day of performance on Experiment 1 and the first day of performance on the control condition. These results indicate that the baboons’ performance was unaffected when the human experimenters were unable to provide subconscious cues to the cup with the larger number. Recall that in this control condition each cup is baited by a different experimenter and neither experimenter knew the quantity of the other’s cup. The baboons were able to discriminate quantity despite this modification of the task procedure, which prevented human cueing.

## Discussion

Eight olive baboons without any prior experience discriminating quantities in experiments were tested on their ability to spontaneously discriminate quantities of food items. The monkeys were able to discriminate small, large, and most importantly, span number pairs, as evidenced by their ability to choose the larger quantity at a frequency significantly above chance. The data show that olive baboons can successfully discriminate quantities, as many other non-human species are known to do. Our data further demonstrate that non-human primates spontaneously discriminate quantities using analog quantity representations that are constrained by ratio and predicted by Weber’s Law.

We tested hypotheses that address the underlying nature of the spontaneous quantity representations of non-human primates. The three hypothetical possibilities we outlined in the Introduction were: (1) object-file numerical representation only, with success occurring only for small numbers, (2) dual incompatible object-file and analog magnitude representation, with success occurring for small and large numbers but not span pairs, and (3) analog magnitude representation only with success dependent on numerical ratio independently of set size. Reviewing the data, we find that the performance of these monkeys is best explained by a single-system analog representation model.

First, monkeys were able to discriminate small, span, and large number pairs presented simultaneously and small and span pairs presented sequentially – numerical discriminations which demonstrably exceed the capacity limit of the object-file system. Failures on sequential large sets were likely due to attentional constraints rather than object-file representations because simultaneous and span discriminations with large values were successful. Anecdotally, we observed that baboons were more distractible over the long sequential trials. This could suggest that failures on large sequential pairs were due to failures of sustained attention. Nonetheless, monkeys were capable of accurate discrimination of span pairs presented sequentially, indicating that they are capable of representing numbers larger than 3 or 4 during sequential presentation. Secondly, the finding that baboons successfully discriminated span pairs indicates that monkeys were not simultaneously using both the analog and object-file systems to perform this task as that hypothesis predicts failure on span pairs (but see Cordes and Brannon, 2008). Finally, monkeys’ performance was ratio-dependent, the diagnostic signature of analog numerical representations. Together, these strands of evidence support the conclusion that the analog magnitude system is the dominant mechanism engaged for spontaneous numerical representation in baboons.

We also investigated the Weber fractions that characterize the numerical sensitivity of individual baboons. The Weber fractions on the first exposure trials were within the range of Weber fractions previously reported for non-verbal numerical discriminations in human children (Halberda and Feigenson, 2008). In fact, the spontaneous quantity judgments of the olive baboons in this study are much like that of 3- and 4-year-old human children, both in terms of discrimination thresholds and in terms of the absence of a capacity limit in numerical discrimination. Prior studies have demonstrated that non-verbal numerical performance in 3- and 4-year-old children also lacks the signatures of “two system” numerical representation (Halberda and Feigenson, 2008; Cantlon et al., 2010).

On the longer exposure experiment with two monkeys, overall performance was significantly above chance for both simultaneous and sequential set presentations and quantity discriminations were modulated by ratio. Weber fractions on the longer exposure experiment were similar to those from the first exposure experiment and so they are also similar to Weber fractions reported for young children. The baboons did not exhibit substantial improvement in overall performance over these dozen or so sessions indicating that learning did not play a major role in baboons’ quantity judgments over across testing. Monkeys’ successful quantity choices during the control condition provides evidence that monkeys did not use subconscious cues by human experimenters to solve the food choice task.

A direct comparison of the baboon data with data previously reported for human infants (Feigenson et al., 2002; Feigenson and Carey, 2003, 2005; Le Corre and Carey, 2007) indicates that non-human primate quantity judgments are not subject to the same constraints as those of human infants. The baboons succeeded at discriminating the numerical pairs that infants have been shown to fail, even on their first exposure to those pairs. Similarly, in the longer exposure experiment baboons performed above chance on numerical pairs that infants fail to discriminate during both sequential and simultaneous presentation trials. These numerical pairs are predicted to elicit failure under an object-file hypothesis due to the capacity limits of the object-file system. Rather than eliciting an object-file signature, baboons’ performance on these pairs exhibited the signature of analog magnitude representation in that performance was modulated by ratio. Again, learning did not appear to affect judgments on these pairs as there was no evidence of significant improvement over the course of testing on these specific pairs. Thus, although some researchers have suggested parallels in numerical cognition between human infants and adult monkeys (e.g., Feigenson et al., 2002), we did not find support for that hypothesis. In fact, as mentioned above, if any parallels can be drawn between the numerical abilities of humans and non-human primates, our data suggest that monkeys might be more similar to 3- and 4-year-old children, at least in terms of their discrimination thresholds (i.e., their Weber fractions) and analog-format numerical representation.

Our results are consistent with prior studies that have argued for spontaneous analog magnitude numerical judgments in many animal species (e.g., Meck and Church, 1983; Brannon and Terrace, 1998; Cordes et al., 2001; Beran, 2007; Cantlon and Brannon, 2007; see Gallistel, 1990 for review). We obtained similar results to these prior studies while also filling a gap in the experimental designs that were used across the studies. In prior studies it remained unclear whether some design aspect – such as experimental training history – might bias non-humans toward behavior consistent with the analog magnitude system. As described in the Introduction, two prior studies reported divergent results: Hauser et al. (2000) found evidence of the object-file system and Beran (2007) found evidence of the analog magnitude system. In both studies, rhesus macaques were presented with two number sets, presented sequentially and placed into boxes, and subjects were tested for their ability to choose the box with the larger quantity. The only difference between those studies was that the Hauser et al. study tested a real-object food choice task with relatively experiment-naive subjects, while the Beran study used a joystick task with experienced subjects. Using a naturalistic food choice task in which experiment-naive subjects were rewarded with whatever amount they chose, we still obtained evidence consistent with the recruitment of a single analog magnitude numerical system. The evidence presented here in support of the analog magnitude system is consistent with the results of Beran (2007) but our experimental design includes important parallels with the design of the Hauser et al. (2000).

The overall success of these experiment-naive baboons on quantitative discriminations of food items indicates that non-human primates spontaneously represent and compare quantities to make adaptive choices. These discriminations can be made over simultaneously or sequentially presented sets of items. The discriminations can also be made over small numerical pairs, large numerical pairs, and pairs that include one small and one large value. Monkeys’ sensitivity in making these discriminations was determined by the ratio between the numerical values of the sets, a signature of analog magnitude representation. The only way to explain the monkeys’ successful performance in these experiments is by appealing to spontaneous quantitative abilities. Our data indicate that these spontaneous quantitative abilities in baboons are inherently analog in nature.

## Conflict of Interest Statement

The authors declare that the research was conducted in the absence of any commercial or financial relationships that could be construed as a potential conflict of interest.
